# Where Do Oncology Patients Seek and Share Health Information? Survey Study

**DOI:** 10.2196/36441

**Published:** 2024-03-25

**Authors:** Eric Freeman, Darshilmukesh Patel, Folasade Odeniyi, Mary Pasquinelli, Shikha Jain

**Affiliations:** 1 College of Medicine University of Illinois at Chicago Chicago, IL United States; 2 Department of Medicine University of Illinois at Chicago Chicago, IL United States

**Keywords:** oncology, social media, patient-physician relationship, patient-physician, patient-provider, cancer, information sharing, information seeking, information behavior, technology access, digital divide

## Introduction

Social media in health care has many benefits, including the dissemination of health information [1] and health promotion [2]. The COVID-19 pandemic has highlighted the benefits of the internet and social media as tools through which individuals can exchange health information. While little is known about oncology patients’ preferences for social media platforms, particularly among minority populations and those in low socioeconomic status communities, some studies have shown its use is linked to the alleviation of patient stress and loneliness, increased feelings of self-efficacy and control of care, and efficient delivery of health information from health practitioners [3]. The study aims to assess where patients from marginalized communities receive a majority of their health care information by surveying patients in a cancer clinic. This study was conducted at the University of Illinois Chicago, which is a public hospital that mainly serves patients from underresourced communities.

## Methods

### Overview

Between March 2021 to June 2021, we administered a 16-item survey (Multimedia Appendix 1) adapted from the National Cancer Institute’s Health Information National Trends Survey (HINTS) [4] to patients scheduled for an oncology visit at the Outpatient Care Center at UI Health. The survey was administered to 145 patients via email and 161 patients in person. Respondents were asked to identify sources used to self-educate about their diagnosis, preferred information source, social media use and preferences, and demographics. We used chi-square tests to assess associations between categorical variables.

### Ethics Approval

This study was approved by the institutional review board at the University of Illinois Chicago and was found to meet the criteria for exemption as defined in the US Department of Health and Human Services Regulations for the Protection of Human Subjects (45 CFR 46.104(d)).

## Results

The demographics of our sample can be found in Table 1. Respondents routinely accessed several forms of health information sources. The top three included their doctor or health care provider (n=274, 89.3%), internet search engines (n=218, 71.2%), and brochures and pamphlets (n=125, 40.7%). However, when directed to choose just one source, 207 (67.4%) chose their doctor or health care provider, while 67 (21.8%) chose internet search engines. The majority of respondents used a smartphone with the internet (n=237, 77.2%), a home desktop or laptop with the internet (n=192, 62.5%), or a tablet with the internet (n=188, 61.2%). However, approximately one-quarter of respondents indicated that they used a mobile phone without internet or a data plan.

We found that the majority of respondents accessed social media in the past year (n=198, 64.7%). Using social media was associated with age (*χ*^2^_3_=18.7; *P*<.001) and sex (Fisher *P*=.001). While respondents primarily used Facebook (n=69, 22.5%), YouTube (n=66, 21.5%), and Instagram (n=25, 8.1%) to receive health information, few shared health information with a medical professional (n=17, 5.5%), and if they did, they primarily used Facebook (n=8, 48.7%).

**Table 1:** Respondent demographics.

		Email (n=145)	In person (n=161)	Statistic (*df*)		*P* value	
Age (years), mean (SD)		60.1 (13.4)	55.3 (15.2)	*t* test: 2.9 (304)		.004	
**Gender, n (%)**					Fisher		.04
	Male	40 (27.6)	48 (29.8)				
	Female	105 (72.4)	107 (66.5)				
	Not reported	0 (0.0)	6 (3.7)				
**Race, n (%)**					Fisher		.26
	Asian	4 (2.8)	6 (3.7)				
	Black or African American	75 (51.7)	85 (52.8)				
	White	49 (33.8)	50 (31.2)				
	Mixed race or biracial	0 (0.0)	5 (3.1)				
	Not reported	17 (11.7)	15 (9.3)				
**Ethnicity, n (%)**					Fisher		<.001
	Hispanic or Latino	18 (12.4)	26 (16.2)				
	Not Hispanic or Latino	125 (86.2)	98 (60.9)				
	Not reported	2 (1.4)	37 (22.9)				
**Primary care, n (%)**					Chi-square: 6.1689 (3)		.10
	Has a primary care provider but has not seen them for over a year	19 (13.1)	22 (13.7)				
	Has a primary care provider not at UI Health, seen in the past year	36 (24.8)	50 (31.1)				
	Has a primary care provider at UI Health, seen in the past year	84 (57.9)	74 (45.9)				
	Does not have a primary care provider	6 (4.1)	15 (9.3)				
**Work status, n (%)**					Fisher		.48
	Full-time paid work or education	31 (21.4)	30 (18.6)				
	Part-time paid work or part-time education	10 (6.9)	9 (5.6)				
	Full-time career/homemaker	3 (2.1)	2 (1.2)				
	Unemployed	40 (27.6)	21 (13)				
	Retired	61 (42.1)	54 (33.5)				
**Current living situation, n (%)**					Fisher		.13
	Alone	36 (24.8)	46 (28.6)				
	With my spouse or partner	56 (38.6)	44 (27.3)				
	With my nonadult children	12 (8.3)	12 (7.5)				
	With family or friends	40 (27.6)	59 (36.7)				
	Homeless	1 (0.7)	0 (0.0)				
**Education, n (%)**					Fisher		<.001
	Less than high school	5 (3.5)	19 (11.8)				
	High school diploma or equivalent	26 (17.9)	48 (29.6)				
	Some college but no degree	32 (22.1)	30 (18.6)				
	Associate’s degree or trade school	15 (10.3)	23 (14.3)				
	Bachelor’s degree	37 (25.5)	26 (16.1)				
	Graduate degree (master’s, MD, JD, PhD)	30 (20.7)	15 (9.3)				
**Annual income (US $), n (%)**					Chi-square: 8.323 (3)		.04
	<15,000	14 (9.7)	31 (19.1)				
	15,000-30,000	18 (12.4)	23 (14.2)				
	30,000	57 (39.3)	44 (27.2)				
	I wish not to answer	56 (38.6)	64 (39.5)				
**Comfort with income, n (%)**					Chi-square: 0.81368 (3)		.85
	Living comfortably on present income	54 (37.2)	56 (35.8)				
	Getting by on present income	58 (40)	63 (38.9)				
	Finding it difficult on present income	26 (17.9)	32 (19.8)				
	Finding it very difficult	7 (4.8)	11 (6.8)				

## Discussion

### Principal Findings

Understanding how patients exchange health information is important to ensure access to accurate information and promote engagement with the health care team. We found that a majority of our patients use social media to find health-related information. However, there continues to be an internet access disparity that can limit patients’ ability to improve their health literacy. As social media engagement is linked to positive patient outcomes, using social media interventions can help us improve oncology patients’ illness experience. While both oncology providers and patients are increasingly using social media as a learning and sharing tool [5], the exact information-seeking behavior of patients with cancer has yet to be fully examined, especially in disadvantaged populations. In the current climate of rampant online medical misinformation, health care workers should find innovative ways to disseminate evidence-based patient-facing information using the platforms most accessed by oncology patients. Our study highlights the need to further explore communication preferences to help develop tailored communication strategies to support underserved patients and their families.

### Limitations

Our study has various limitations. This study was a single clinic, single institution study with a relatively small sample size. Additionally, our patient population was older, which could have influenced preferred social media platforms.

## Appendix

Multimedia Appendix 1 Social media survey.

## Abbreviations

HINTS: Health Information National Trends Survey

## Data Availability

The data sets generated or analyzed during this study are available from the corresponding author upon reasonable request.
