# Supplementary material for: Where Do Oncology Patients Seek and Share Health Information? Survey Study
Source: J Med Internet Res. 2024 Mar 25;26:e36441. doi: 10.2196/36441 (PMC11002738; doi:10.2196/36441)
Supplement: Multimedia Appendix 1 [file jmir_v26i1e36441_app1.docx]

# **Appendix 1. Social Media Survey**

**Social Media Engagement and Information Seeking Behavior**

People get information from many different sources. It is important for your doctors and the care team to know the best way to get accurate and relevant health information to you.

Your participation in this study will help us learn more about sources and social media platforms patients like you use to learn about health.

| **Patient Name:** |  |
| --- | --- |
| **Patient E-mail:** |  |
| **MRN:** |  |
| **Age:** |  |
| **Gender:** | - Male - Female |
| **Race (Mark ALL that apply):** | - American Indian and Alaska Native - Asian - Black or African American - Native Hawaiian and Other Pacific Islander - White - Other (please specify): |
| **Ethnicity** | - Hispanic or Latino - Not Hispanic or Latino |
| **Zip code:** |  |
| **Primary language spoken in home** | - English - Cantonese - Mandarin - Polish - Spanish - Other (please specify)ο: |
| **Cancer Type** | - Bladder cancer - Bone cancer - Breast cancer - Cervical cancer (cancer of the cervix) - Colon cancer - Endometrial cancer (cancer of the uterus) - Head and neck cancer - Leukemia/Blood cancer - Liver cancer - Lung cancer - Lymphoma (Hodgkin’s) - Lymphoma (Non-Hodgkin’s) - Melanoma - Oral Cancer - Ovarian cancer - Pancreatic cancer - Pharyngeal (throat) cancer - Prostrate cancer - Rectal cancer - Renal (kidney) cancer - Skin cancer, non-melanoma - Stomach cancer - Other (please specify): |

1. **Imagine that you had a strong need to get information about your illness. What sources would you use?** (**Mark ALL that apply**)

| - Books | - Library |
| --- | --- |
| - Brochures, pamphlets, etc | - Magazines |
| - Doctor or health care provider | - Newspapers - Non-traditional medical practitioner (ie chiropractor, homeopathic practitioner, traditional healer) |
| - Family | - Podcasts |
| - Friend/ Co-worker | - Radio |
| - Healthcare non-profit organization (American Cancer Society, American Heart Association, etc.) | - Television - Other (please specify): |
| - Hospital website - Internet search engine (Google, Yahoo, etc) |  |

1. **Now, imagine that you had a strong need to get information about your illness, but you can only choose ONE source. Where would you go first?** (**Choose only one**)

| - Books | - Library |
| --- | --- |
| - Brochures, pamphlets, etc | - Magazines |
| - Doctor or health care provider | - Newspapers - Non-traditional medical practitioner (ie chiropractor, homeopathic practitioner, traditional healer) |
| - Family | - Podcasts |
| - Friend/ Co-worker | - Radio |
| - Healthcare non-profit organization (American Cancer Society, American Heart Association, etc.) | - Television - Other (please specify): |
| - Hospital website - Internet search engine (Google, Yahoo, etc) |  |

1. **Do you have access to these technologies?** (**Mark ALL that apply**)

| - Tablet with Internet or Wi-Fi (e.g., iPad, Samsung Galaxy) | - Home desktop computer/ laptop with internet or Wi-Fi |
| --- | --- |
| - Work desktop computer/ laptop with internet or Wi-Fi | - Other (please specify): |
| - Mobile phone without internet or data plan | - No computer or tablet |
| - Smart phone (e.g., iPhone, Android) with internet | - No phone |

**Sometimes people use the Internet to connect with other people online through social networks like Facebook or Twitter. This is often called “social media.”**

1. **Do you use any social media platform? (Facebook, Twitter, Instagram, etc)**

| - No | - Yes |
| --- | --- |

1. **In the last 12 months, which social media platforms have you used?** (**Mark ALL that apply**)

| - Groupme | - Tiktok |
| --- | --- |
| - Facebook | - Twitter |
| - Instagram | - WeChat |
| - Linkedin | - YouTube |
| - Pinterest | - Other (please specify): |
| - Reddit | - I don’t use social media |
| - Snapchat |  |

1. **In the last 12 months, have you received any health information from any of the following social media platforms?** (**Mark ALL that apply**)

|  | **No** | **Yes** |
| --- | --- | --- |
| Groupme |  |  |
| Facebook |  |  |
| Instagram |  |  |
| Linkedin |  |  |
| Pinterest |  |  |
| Reddit |  |  |
| Snapchat |  |  |
| Tiktok |  |  |
| Twitter |  |  |
| WeChat |  |  |
| YouTube |  |  |
| Other (please specify): |  |  |

1. **In the last 12 months,** **have you shared health information on any of the following social networking sites?** (**Mark ALL that apply**)

|  | **No** | **Yes** |
| --- | --- | --- |
| Groupme |  |  |
| Facebook |  |  |
| Instagram |  |  |
| Linkedin |  |  |
| Pinterest |  |  |
| Reddit |  |  |
| Snapchat |  |  |
| Tiktok |  |  |
| Twitter |  |  |
| WeChat |  |  |
| YouTube |  |  |
| Other (please specify): |  |  |

1. **In the past 12 months, have you used social media (e.g., Facebook, Twitter etc.) to exchange medical information with a health care professional?**

| - No | - Yes |
| --- | --- |

**Now we would like to learn a little about you**

1. **Primary Care:**

| - I have a primary care provider but have not seen them for over a year |
| --- |
| - I have a primary care provider not at UI Health that I have seen in the past year |
| - I have a primary care provider at UI Health that I have seen in the past year |
| - I do not have a primary care provider |

1. **Work status**

| - In full-time paid work | - Full-time career/homemaker |
| --- | --- |
| - In part-time paid work | - On leave/out of work due to illness |
| - In full-time education | - Out of work due to COVID-19 pandemic |
| - In part-time education | - Unemployed |
|  | - Retired - Other (please specify): |

1. **Including yourself, how many people age 18 or older live in your household?:**
2. **How many children under the age of 18 live in your household?:**
3. **How would you describe your current living situation? I live…**

| - Alone | - With my grandchildren |
| --- | --- |
| - With my spouse or partner | - With friends |
| - With my adult children (18 years and up) | - With my parents |
| - With my non-adult children (under 18 years) | - Other (please specify): |

1. **Your Highest Level of Education:**

| - Less than high school diploma |
| --- |
| - High school diploma or GED |
| - Some college but no degree |
| - Associate’s Degree (for example AA, AS) |
| - Bachelor’s Degree (for example BA, AB, BS) |
| - Master’s Degree (for example MA, MS) |
| - Doctoral or Professional Degree (for example MD, DDS, PhD, EdD) - Other (please specify): |

1. **Thinking about members of your family living in this household, what is your combined annual income, meaning the total pre-tax income from all sources earned in the past year?**

| - <$15,000 - $15,000 to $30,000 - >$30,000 |
| --- |
| - I wish not to answer |
|  |

1. **Which one of these comes closest to your own feelings about your household’s income?**

| - Living comfortably on present income - Getting by on present income | - Finding it difficult on present income - Finding it very difficult on present income |
| --- | --- |

Thank you for completing the survey. Your participation is greatly appreciated.
